# Patient-oriented educational Sports Medicine YouTube videos in Arabic have higher view counts in the Middle East and North Africa than their English versions

**DOI:** 10.1007/s00264-023-05970-z

**Published:** 2023-09-13

**Authors:** Theodorakys Marín Fermín, Ashraf T. Hantouly, Ayyoub A. Al-Dolaymi, Bruno C. R. Olory, Elisabet Hagert, Emmanouil T. Papakostas, Bashir A. Zikria

**Affiliations:** 1 Centro Médico Profesional Las Mercedes, Av. Principal de Las Mercedes, piso 3, consultorio 37, Caracas, Venezuela; 2 Orthopedics Department, Surgical Specialty Center, Hamad General Hospital, Hamad Medical Corporation, Doha, Qatar; 3 Medicine College, University of Anbar, Anbar, Iraq; 4 Aspetar Orthopaedic and Sports Medicine Hospital, Doha, Qatar

**Keywords:** Communication barrier, Social media, Patient preferences, Culture gap, Engagement

## Abstract

**Purpose:**

The present study aims to assess the impact of the local language on the view count of patient-oriented educational Sports Medicine videos in an Orthopaedic and Sports Medicine Hospital in the Middle East and North Africa.

**Methods:**

An observational study on English and Arabic versions of Aspetar’s YouTube channel patient-oriented educational video series was conducted in February 2023, comparing the view count and viewer characteristics. Included videos were posted either simultaneously or in English version first, in both languages, and shared on the same media platforms. Collected data of interest included video title, view count in each language, age and sex of the viewers, location, and traffic source.

**Results:**

Eleven videos of the patient-oriented educational video series were included in the present study. Except for one, the view count was significantly higher in the Arabic version of all 11 videos (minimum sevenfold, *P* = 0.03). Viewers were predominantly males (73.9%) and between 18 and 44 years old (81.1%). Eleven out of 19 countries of the Middle East and North Africa region [11] were among the viewers’ top 20 countries. Traffic sources included YouTube search (45.9%), YouTube suggested videos (17%), external sources (14.4%), YouTube browse features (8.5%), and YouTube advertising (6%).

**Conclusion:**

Patient-oriented educational Sports Medicine videos in Arabic yield higher view counts than their English version in young adult viewers from 11 countries in the Middle East and Africa among the top 20. Content creation on languages with limited online representation could effectively reach the targeted population by breaking language barriers.

## Introduction

Patient-oriented educational videos are a growing and widespread tool in healthcare [1]. They are an attractive alternative to surgery, rehabilitation, or medical advice leaflets with better visual value [1]. Recent studies have suggested that patient-oriented educational videos can improve patient experience (reducing anxiety and stress) and potentially benefit outcomes of medical care [2]. Moreover, high-quality videos can empower patients by improving their understanding of their medical condition, making them more confident and involved in decision-making [1, 3].

YouTube is the second-largest social network, with 1.9 billion users, with young adults among their most common users [3, 4]. Despite the concerns about the quality of its content, it has a global reach, with 80% of its users outside the USA [3, 5, 6]. Studies suggest that almost half of the patients search online about their condition before orthopaedic consultations and 42% after it [7]. However, for Arabic-speaking people, only 3% of the internet content is available in their language [8].

Although the Middle East is among the regions with the most English speakers, their proficiency level is very low [9]. Considering language can significantly impact healthcare access and quality, studies on overcoming this barrier are of utmost importance in this population [10]. Thus, the present study aims to assess the impact of the local language on the view count of patient-oriented Sports Medicine educational videos in an Orthopaedic and Sports Medicine Hospital in the Middle East and North Africa.

## Methods

An observational study on English and Arabic versions of Aspetar’s YouTube channel patient-oriented educational Sports Medicine video series was conducted in February 2023, comparing the view count and viewer characteristics. Included videos were posted either simultaneously or in English version first, in both languages, and shared on the same media platforms. The videos were produced by the Hospital’s Marketing Department in collaboration with expert healthcare professionals and translated into modern standard Arabic by a certified translator. Collected data of interest included video title, view count in each language, age and sex of the viewers, geographic location, and traffic source.

### Statistical analysis

Descriptive statistics were used to quantify view count and viewer characteristics. An unpaired *t*-test was assessed to compare the view count of English and Arabic videos. All statistical tests were performed with SPSS V26.0 was used for analysis (IBM Corporation, Armonk, NY). *P*-values < 0.05 were considered to indicate statistical significance.

## Results

**Fig. 1:**
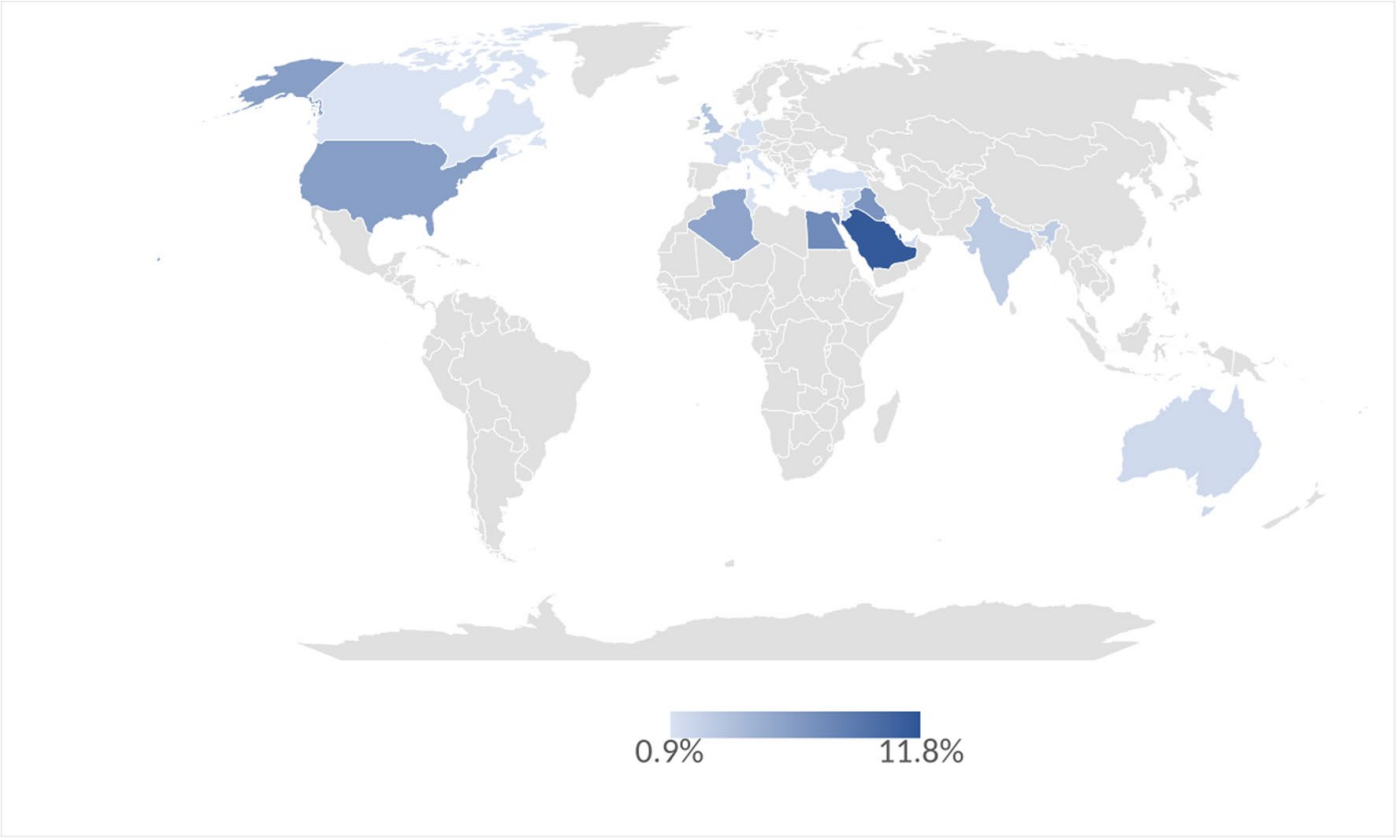
World map viewers’ percentage distribution in the top 20 countries

Eleven videos of the patient-oriented educational Sports Medicine video series were included in the present study (Table 1). Except for one, the view count was significantly higher in the Arabic version of all 11 videos (minimum sevenfold, *P* = 0.03). Viewers were predominantly males (73.9%) and between 18 and 44 years old (81.1%) (Table 2). Eleven out of 19 countries of the Middle East and North Africa region [11] were among the viewers’ top 20 countries (Table 3, Fig. 1). Traffic sources included YouTube search (45.9%), YouTube suggested videos (17%), external sources (14.4%), YouTube browse features (8.5%), and YouTube advertising (6%).

**Table 1:** View count of English and Arabic versions of Aspetar’s YouTube channel patient educational Sports Medicine video series in February 2023

Video title	Views—English versión*	Views—Arabic versión*
Most common types of hand and wrist injuries	8.300	631.000
Groin pain symptoms, types, prevention, treatments, and return to play	73.000	524.000
Meniscus tear in the knee: symptoms, causes, and treatments	5.100	308.000
ACL injuries diagnosis, causes, treatment options, and return to play	1.600	118.000
Plantar fasciitis symptoms, causes, and treatments	3.700	107.000
Tennis elbow symptoms, causes, and treatments	490	14.000
All you need to know about hernia in sports	2.300	21.000
Common sports injuries involving the shoulder	5.900	51.000
Sudden cardiac arrest in sports	2.300	15.000
What are muscle strains?	16.000	15.000
How to choose a good exercise shoe?	3.900	42.000
Total	122.590	1.846.000

*Values were rounded down to the nearest ten

**Table 2:** Viewers characteristics

	Percentage
Sex	
Male	73.9%
Female	26.2%
Age group	
13–17 years	3.1%
18–24 years	25.5%
25–34 years	35.6%
35–44 years	20%
45–54 years	9.3%
55–64 years	4.4%
65 years and more	2.2%

**Table 3:** Viewers’ location—top 20 countries

*N*	Countries	Percentage
1	Qatar	11.8%
2	Saudi Arabia	11.4%
3	Egypt	7.6%
4	Iraq	6.9%
5	USA	6.1%
6	Algeria	5.6%
7	Morrocco	4.7%
8	UK	3.2%
9	India	2.6%
10	United Arab Emirates	1.6%
11	France	1.6%
12	Australia	1.6%
13	Kuwait	1.5%
14	Syria	1.3%
15	Jordan	1.3%
16	Turkey	1.1%
17	Germany	1.1%
18	Italy	1.0%
19	Tunisia	1.0%
20	Canada	0.9%
	Top 20 countries total	73.9%
	Others	26.1%

## Discussion

The main finding of the present study is that patient-oriented educational Sports Medicine video series in Arabic yield higher view counts than their English version, including young adult viewers from 11 countries in the Middle East and Africa among the top 20.

Given the limited online content in Arabic, patient-oriented educational videos are of great value. Our results show that almost half of the traffic to the videos are directly from YouTube search, suggesting the use of Arabic keywords during browsing is strongly related to the retrieval of the videos of our study among the targeted population. Social media use among sports surgeons is growing, especially among knee and shoulder specialists, mostly on academic platforms such as LinkedIn and ResearchGate [12, 13]. On the other hand, YouTube has been identified as the media platform with the least surgeons’ presence, probably due to the time demands in video editing for content creation [13]. Ironically, patients show great interest in educational and medical facts videos from their sports surgeons’ social media [14].

Most of the literature on YouTube patient-oriented educational videos has targeted content quality and reliability. Springer et al. [15] highlighted the poor information quality, reliability, and accuracy among 140 YouTube videos on anterior cruciate ligament rehabilitation and return to sport. Abed et al. [5] analysis of 50 videos revealed poor transparency, reliability, and content quality on patellar dislocation. Likewise, Springer et al. [16] analysis showed poor quality, accuracy, and reliability of the information on 102 postoperative rotator cuff rehabilitation videos. Similarly, Matzko et al. [17] found low overall quality and reliability scores among 50 SLAP tears videos. However, none of the tools assessing such quality evaluate the implemented language [18, 19].

Other researchers have assessed quality and reliability in their local languages, including Arabic, with comparable outcomes [20–23]. However, few have assessed local language and video engagement. Jenkin Sy et al. [24] evaluated the engagement and content of YouTube videos on hydrocephalous in three groups: (1) most viewed Filipino-language videos, (2) most viewed English-language videos, and (3) same-age English-language videos matched to the first group based on upload date. They found that Filipino videos had a higher median number of likes and comments and were more likely to host discussions on treatment costs and to solicit donations but poor video quality. Although assessing the video quality was beyond the scope of our study, our findings show similar results, highlighting the impact of native language on the targeted population. YouTube videos in languages with limited available content seem to impact patient and caregiver engagement greatly; thus, creating content in such languages or translating them represents a great advantage for regional diffusion.

This study is not free of limitations, but those are inherent to the metrics assessed by YouTube Analytics. Future studies should aim to develop content creation guidelines for physicians and institutions to improve video content quality and a registry of verified videos for patient education.

## Conclusion

Patient-oriented educational Sports Medicine videos in Arabic yield higher view counts than their English version in young adult viewers from 11 countries in the Middle East and Africa among the top 20. The results show that modern standard Arabic is not a barrier to explaining medical terms to the public. Content creation on languages with limited online representation could effectively reach the targeted population by breaking language barriers.

## Data Availability

The data underlying this article are available in the article and its online supplementary material.
